# Expression Profiles of PIWIL2 Short Isoforms Differ in Testicular Germ Cell Tumors of Various Differentiation Subtypes

**DOI:** 10.1371/journal.pone.0112528

**Published:** 2014-11-10

**Authors:** Ildar V. Gainetdinov, Yulia V. Skvortsova, Elena A. Stukacheva, Oksana S. Bychenko, Sofia A. Kondratieva, Marina V. Zinovieva, Tatyana L. Azhikina

**Affiliations:** Shemyakin-Ovchinnikov Institute of Bioorganic Chemistry, Russian Academy of Sciences, Moscow, Russia; Shanghai Jiao Tong University School of Medicine, China

## Abstract

PIWI family proteins have recently emerged as essential contributors in numerous biological processes including germ cell development, stem cell maintenance and epigenetic reprogramming. Expression of some of the family members has been shown to be elevated in tumors. In particular, PIWIL2 has been probed as a potential neoplasia biomarker in many cancers in humans. Previously, PIWIL2 was shown to be expressed in most tumours as a set of its shorter isoforms. In this work, we demonstrated the presence of its 60 kDa (PL2L60A) and 80 kDa (PL2L80A) isoforms in testicular cancer cell lines. We also ascertained the transcriptional boundaries of mRNAs and alternative promoter regions for these PIWIL2 isoforms. Further, we probed a range of testicular germ cell tumor (TGCT) samples and found PIWIL2 to be predominantly expressed as PL2L60A in most of them. Importantly, the levels of both PL2L60A mRNA and protein products were found to vary depending on the differentiation subtype of TGCTs, i.e., PL2L60A expression is significantly higher in undifferentiated seminomas and appears to be substantially decreased in mixed and nonseminomatous TGCTs. The higher level of PL2L60A expression in undifferentiated TGCTs was further validated in the model system of retinoic acid induced differentiation in NT2/D1 cell line. Therefore, both PL2L60A mRNA and protein abundance could serve as an additional marker distinguishing between seminomas and nonseminomatous tumors with different prognosis and therapy approaches.

## Introduction

Recently, PIWI family proteins [1], [2] have come to light as a new set of players in transcriptional and post-transcriptional regulation of gene expression [3]–[10]. Their contribution has been shown to be significant for processes ranging from protection of genome against transposon activity during spermatogenesis [11], [12] and stem cell maintenance [13], [14] to somatic regulation and establishment of epigenetic states [15]–[18].

Although their expression is typically confined to germ cells, several members of this family turned out to be upregulated across tumors [19], [20]. Among them, PIWIL2 (MILI in mice, HILI in humans) has been repeatedly proposed as a potential marker for cancers of various origin [21]–[32]. Several groups of researchers have worked to demonstrate its regulational contribution into neoplasia through different signaling pathways [33]–[36].

However, the picture has become increasingly complex due to several reports on PIWIL2 downregulation in some tumors [37], [38]. Furthermore, in human soft tissue sarcoma lower *PIWIL2* mRNA expression was significantly associated with a worse prognosis for patients [39]. Finally, PIWIL2 knockdown in murine bone marrow mesenchymal stem cells has been shown to enhance cell proliferation and decrease expression of tumor suppressors [40].

Another notable fact is the existence of multiple protein isoforms of PIWIL2 [35]. Indeed, Ye *et al.* [35] found the expression of PIWIL2 in most cancer cell lines assayed and precancerous stem cells to be represented almost exclusively by its 60 kDa variant (PL2L60). In this view, PIWIL2 multi-domain structure should be taken into account. Specifically, N-terminal sequence of PIWIL2 harbors conservative arginine methylation sites which are essential for binding TUDOR-domain containing proteins [41]–[44]. Also, PAZ and PIWI domains are a single-stranded nucleic acid-binding and an RNase H motifs, respectively [45], [46]. One could imply that variants of PIWIL2 with various segments of the full-length protein might possess different properties and affinities. Similarly, ratio of PIWIL2 isoforms present in the cell may contribute into cellular processes where PIWIL2 is involved.

In this work, we attempted to expand the knowledge on the presence of different PIWIL2 isoforms in various *testicular* cancer cell lines and *testicular* germ cell tumors (TGCTs), as well as elicit correlations of PIWIL2 isoforms expression with neoplastic profiles and clinical manifestations of TGCTs. Surprisingly, we found the expression of PIWIL2 isoforms to be different between TGCTs of various differentiation stages. Additionally, we managed to confirm these findings using retinoic acid induced cell culture differentiation. Finally, we established alternative transcription start and polyadenylation sites for PIWIL2 short isoforms in testicular cancer cell lines.

## Methods and Materials

### Ethics Statement

42 samples of testicular germ cell tumors and 1 sample of testicular parenchyma (normal testis) were obtained from orchiectomy specimens with testicular germ cell tumors under non-neoplastic conditions. Representative samples were immediately frozen in liquid nitrogen. The sampling was made with a written consent of the patients according to the federal law and approved by the ethical committees of the Shemyakin-Ovchinnikov Institute of Bioorganic Chemistry of the Russian Academy of Sciences (SIOBC) and Blokhin Cancer Research Center of the Russian Academy of Medical Sciences (BCRC). Institutional Review Boards of both SIOBC and BCRC approved the protocol for this study after reviewing the informed consent and patient information forms.

### Cell lines and retinoic acid induced differentiation

Cell lines used in experiments included TERA1 (ATCC HTB-105, testicular embryonal carcinoma [47]), NT2/D1 (ATCC CRL-1973, pluripotent testicular embryonal carcinoma [48]), A549 (ATCC CCL-185, lung carcinoma [49]), T-47D (ATCC HTB-133, mammary gland ductal carcinoma [50]), HeLa (ATCC CCL-2, cervical adenocarcinoma [51]), Raji (ATCC CCL-86, Burkitt's lymphoma [52], [53]), Jurkat (ATCC TIB-152, acute T-cell leukaemia [54]), NGP-127 (neuroblastoma, [55]), IMR-32 (ATCC CCL-127, neuroblastoma [56]), Daudi (ATCC CCL-213, Burkitt's lymphoma [57]), A-431 (ATCC CRL-1555, epidermoid carcinoma [49]), HEK-293 (ATCC CRL-1573, embryonic kidney [58]), Hep G2 (ATCC HB-8065, hepatocellular carcinoma [59]) and HL-60 (ATCC CCL-240, acute promyelocytic leukemia [60]).

Cells cultures were purchased by Shemyakin-Ovchinnikov Institute of Bioorganic Chemistry from ATTC (USA) and grown in DMEM/F12 (1∶1) (Invitrogen, USA) supplemented with 10% FCS (Invitrogen, USA). NGP-127 cell line (neuroblastoma) was kindly provided by Paul S. Meltzer (NHGRI, NIH, Bethesda, MD, USA) and was grown as described previously [61].

Retinoic acid (RA) induced differentiation of TERA1 and NT2/D1 cell lines was conducted in the presence of 10 mkM RA (Sigma, USA) [62], [63]. Cell cultures with no treatment were used as controls. Cells were harvested before the start of RA induction at Day 0 and at Days 1, 2 for both cell lines and at Day 3 for TERA1 only after the addition of RA. Biological duplicates were used to ensure reproducibility.

### Western blotting

Western blot analyses were carried out using crude cell lysates of cell lines or testicular cancer samples heated for 5 min at 95°C in 2x SDS sample buffer (100 mM Tris-HCl, pH 6.8, 4% SDS, 0.2% Bromophenol Blue, 20% glycerol, 200 mM DTT). Proteins were separated in 10% polyacrylamide gel and transferred to Hybond-P membrane (GE Healthcare, UK).

Treatment with primary antibodies listed in Table S1 was followed by the addition of either secondary anti-rabbit IgG HRP-linked antibody (Cell Signaling, USA, #7074S) or secondary anti-mouse IgG HRP-linked antibody (Cell Signaling, USA, #7076S). The membrane was visualized using the Immun-Star HRP Chemiluminescent kit (Bio-Rad, USA) and bands were detected either in VersaDoc MP4000 imager (Bio-Rad, USA) or on X-ray film. Technical duplicates were used to ensure reproducibility.

### siRNA assay

TERA1 and NT2/D1 cell lines were reverse transfected with custom synthesized siRNA duplexes (DNA synthesis, Moscow, Russia) in presence of Lipofectamin RNAiMAX (Life Technologies, USA) as recommended by the manufacturer. Biological duplicates were used to ensure reproducibility. The sequences of siRNA duplexes were as follows: scrambled RNA -GCAUGAGCGACCACUCCUAdTdT and UAGGAGUGGUCGCUCAUGCdTdT, siRNA 1 - CCAUUGGCAGAACACGUCCdTdT and GGACGUGUUCUGCCAAUGGdTdT, siRNA 2 - CUUCCUUAACCCAGUUUAGdTdT and CUAAACUGGGUUAAGGAAGdTdT.

### RACE

Total RNA extraction and purification from TERA1 and NT2/D1 cell lines were performed according to Sambrook *et al.* [64]. 5′- and 3′-RACE experiments were conducted using SMART RACE cDNA Amplification Kit (Clontech, USA). Gene specific primers utilized in the experiment are listed in Tables S2 and S3. Biological duplicates were used to ensure reproducibility.

### RT-qPCR and RT-PCR

Total RNA extraction and purification from TERA1 and NT2/D1 cell lines, testicular cancer and normal testis samples were performed as described above. First strand cDNA synthesis was carried out with the random hexanucleotide primer (Promega, USA) and MintReverse Transcriptase (Evrogen, Russia) according to the manufacturers' protocols. For RT-qPCR, reactions were performed using qPCRmix-HS SYBR system (Evrogen, Russia) on Lightcycler 480 (Roche, USA) in accordance with the manufacturers' instructions. DNA fragments were amplified for 40 cycles of 95°C for 20 s, 60°C for 20 s, 72°C for 20 s. Relative level of mRNA was quantified with 18S rRNA serving as the reference. Technical triplicates were used to ensure reproducibility. For RT-PCR, reactions were performed using Encyclo Polymerase Mix (Evrogen, Russia) on Biorad DNAEngine PTC (Biorad, USA). DNA fragments were amplified for 35 cycles of 95°C for 20 s, 60°C for 20 s, 72°C for 3 min. Primer pairs used in amplification are listed in Table S4.

### Luciferase reporter vectors, transfection and reporter gene assay

Reporter vectors were constructed with the genomic sequences upstream of each transcription start site detected in RACE experiments. Each putative promoter region was PCR amplified using human genomic DNA extracted from normal testis sample according to Sambrook *et al.* [64]. PCR primers contained NheI restriction enzyme recognition site (Table S5) to facilitate further cloning of the amplification product into pGL4.10 (Promega, USA) upstream of the reporter firefly (*Photinus pyralis*) luciferase gene. Transfections were performed using Lipofectamine 2000 (Invitrogen, USA) as recommended by the manufacturer. Cells were lyzed 24 hours after the transfection and the activity of both firefly and *Renilla reniformis* luciferases was assessed using DualLuciferase Reporter Assay System (Promega, USA) and Tecan GENios Pro Luminometer (MTX Lab Systems, USA) according to the manufacturers' protocols. Biological and technical duplicates were used to ensure reproducibility.

### PIWIL2 control isoforms cloning and transient transfection in HEK293 cell line

RNA extraction and purification from the normal testis sample were performed according to Sambrook *et al.* [64]. First strand cDNA synthesis was carried out with the random hexanucleotide primer (Promega, USA) and MintReverse Transcriptase (Evrogen, Russia) according to the manufacturers' protocols. cDNA for each PIWIL2 control isoform was PCR amplified with primers containing EcoRI and NotI restriction enzymes recognition sites (Table S6) to facilitate further cloning of the amplification product into pCI vector (Promega, USA). Transfections were performed using Lipofectamine 2000 (Invitrogen, USA) as recommended by the manufacturer. Cells were lyzed 48 hours after the transfection and probed by Western blot analysis. Biological duplicates were used to ensure reproducibility.

## Results

### Patterns of PIWIL2 isoforms expression in different cell lines are not uniform

The 60 kDa isoform of PIWIL2 (PL2L60, PIWIL2-like 60 kDa protein) was described by Ye *et al.* [35] and subsequently proposed as a putative marker of neoplasia expressed in the majority of cell lines studied. Since Ye *et al.* assumed PL2L60 to span exons 11 to 23 [35], we used a commercially available polyclonal antibody specific to exons 14–16 of PIWIL2 (14–16ex PIWIL2 antibody, HPA029345, Sigma, USA). We assayed various cell lines of germ and somatic origin including TERA1 (testicular embryonal carcinoma), NT2/D1 (pluripotent testicular embryonal carcinoma), A549 (lung carcinoma), T-47D (mammary gland ductal carcinoma), HeLa (cervical adenocarcinoma), Raji (Burkitt's lymphoma), Jurkat (acute T-cell leukaemia), NGP-127 (neuroblastoma), IMR-32 (neuroblastoma), Daudi (Burkitt's lymphoma), A-431 (epidermoid), HEK-293 (embryonic kidney), Hep G2 (hepatocellular carcinoma) and HL-60 (acute promyelocytic leukemia).

**Figure 1:**
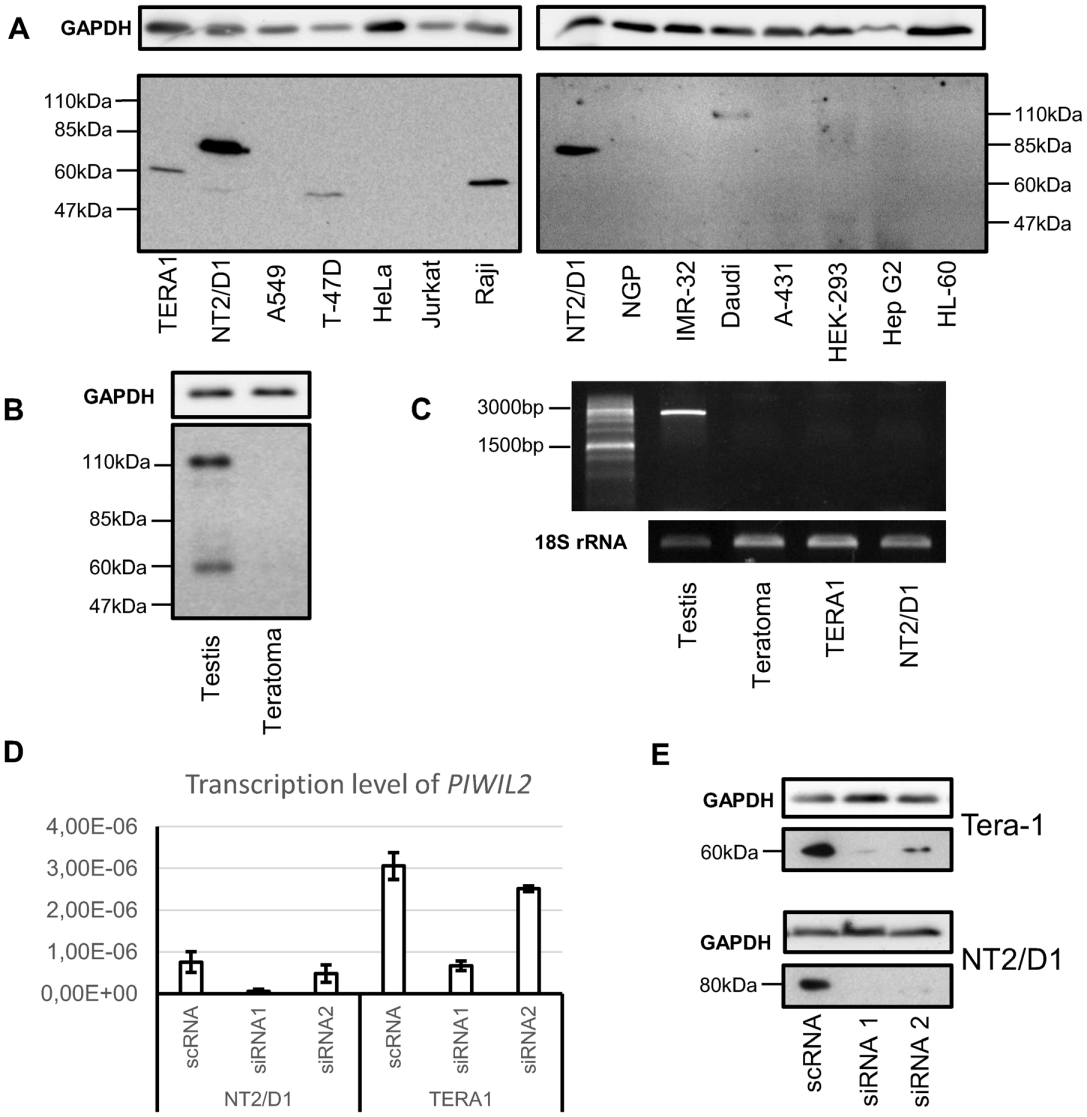
Specificity of 14-16ex PIWIL2 antibody. Western blot analyses of a panel of cell lines (**A**) and testis with its adjacent tumor (**B**) with an antibody specific to exons 14–16 of PIWIL2. RT-PCR on cDNA from testis, teratoma, TERA1 and NT2/D1 cell lines with primers for exons 2–23 of *PIWIL2* mRNA (**C**). Results of siRNA assay: transcription level of *PIWIL2* mRNA (exons 8–9) relative to 18S rRNA (**D**) and PIWIL2 protein isoforms in TERA1 and NT2/D1 (**E**).

The results we obtained were not uniform across cell lines: presence of 60 kDa PIWIL2 isoform was only observed in TERA1, T47D and Raji, as well as 80 kDa variant in NT2/D1 and the full-length PIWIL2 in Daudi cells (Fig. 1A). Importantly, since we used an antibody which is different from the one in the study by Ye *et al.* [35], we were unable to directly compare our findings. Therefore, we termed the detected 60 kDa and 80 kDa isoforms PL2L60A and PL2L80A, respectively. We also performed Western blotting with a sample of testis and its adjacent tumor (teratoma) to show that 14–16ex PIWIL2 antibody is able to detect the full-length PIWIL2 protein (Fig. 1B). Furthermore, RT-PCR with cDNA from normal testis, teratoma, TERA1 and NT2/D1 cell lines confirmed the absence of mRNA for the full-length PIWIL2 in all samples except testis (Fig. 1C).

To further validate the specificity of 14–16ex PIWIL2 antibody, we designed siRNA duplexes to exon 15. PIWIL2 mRNA expression was knocked down in TERA1 and NT2/D1 expressing PL2L60A and PL2L80A, respectively (Fig.1D). Both mRNA and protein level were significantly reduced providing additional evidence for the specificity of 14–16ex PIWIL2 antibody (Fig. 1E).

### Identification of transcriptional boundaries of PL2L60A in TERA1 and PL2L80A in NT2/D1

In order to identify transcription start and polyadenylation sites for mRNAs corresponding to PL2L60A and PL2L80A, we used TERA1 and NT2/D1 cell lines, respectively. Based on the GRCh37/hg19 genome assembly, PIWIL2 specific primers were designed (Tables S2 & S3) and 5′/3′-RACE experiments were conducted.

**Figure 2:**
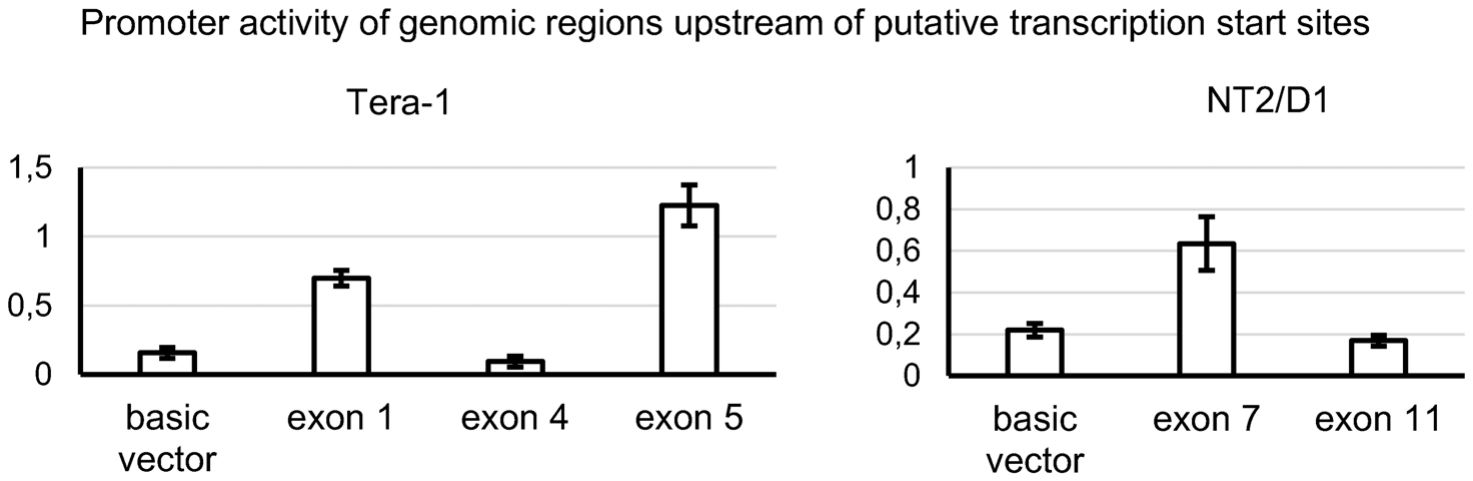
Promoter activity of genomic regions upstream of putative transcription start sites. Promoter activity measured as a ratio of firefly (*Photinus pyralis*) luciferase to *Renilla reniformis* luciferase luminescence in TERA1 and NT2/D1 cell lines for genomic regions upstream of each putative transcription start sites mapped in 5′-RACE experiments. Basic vector (negative control) is included.

We identified possible transcription start sites for PL2L60A (TERA1) in exons 1, 4 and 5 and for PL2L80A (NT2/D1) in exons 7 and 11 (Table 1). In order to validate them, we cloned appropriate upstream genomic regions (Table S5) into firefly (*Photinus pyralis*) luciferase reporting constructs. The constructs were transfected into TERA1 and NT2/D1 cells along with pRL-TK reference vector coding for *Renilla reniformis* luciferase and relative luciferase activity was obtained (Fig. 2).

**Table 1:** Results of 5′ and 3′-RACE experiments in TERA1 and NT2/D1 cell lines.

Cell line	5′/3′-RACE	Coordinate on chromosome 8 in GRCh37/hg19 (exon number, position in PIWIL2 cDNA NM_018068.3)
TERA1	5′RACE	22132875 (exon 1, 66), 22139056 (exon 4, 551), 22140703 (exon 5, 730)
	3′RACE	22167494 (exon 15, 1856), 22179520 (exon21a in intron 20, 2552+162)
NT2/D1	5′RACE	22145061 (exon 7, 914), 22161534 (exon 11, 1330), 22161606 (exon 11, 1402)
	3′RACE	22165574 (exon 14, 1822), 22213423 (exon 23, 3471), 22213601 (exon 23, 3637)

Noticeable promoter activity was only observed in genomic regions upstream of putative transcription start sites in exons 1 and 5 in TERA1 (for PL2L60A) and exon 7 in NT2/D1 (for PL2L80A). Furthermore, these genomic areas coincided with peaks of histone marks for active promoter (histone H3 lysine 4 trimethylation) and regulatory regions (histone H3 lysine 4 monomethylation and histone H3 lysine 27 acetylation), as well as DNaseI hypersensitivity clusters and increased density of binding sites for both basal and pathway/tissue-specific transcription factors from ENCODE (Fig. S1) [65], [66].

3′-RACE experiments identified the annotated polyadenylation site only in NT2/D1 cell line (Table 1). Moreover, in NT2/D1, additional putative transcription termination sites were found in exon 14 (30 bp downstream of AAUGAA polyadenylation signal [67], [68]) and exon 23 (upstream of a GU-rich stretch [69], [70]).

**Figure 3:**
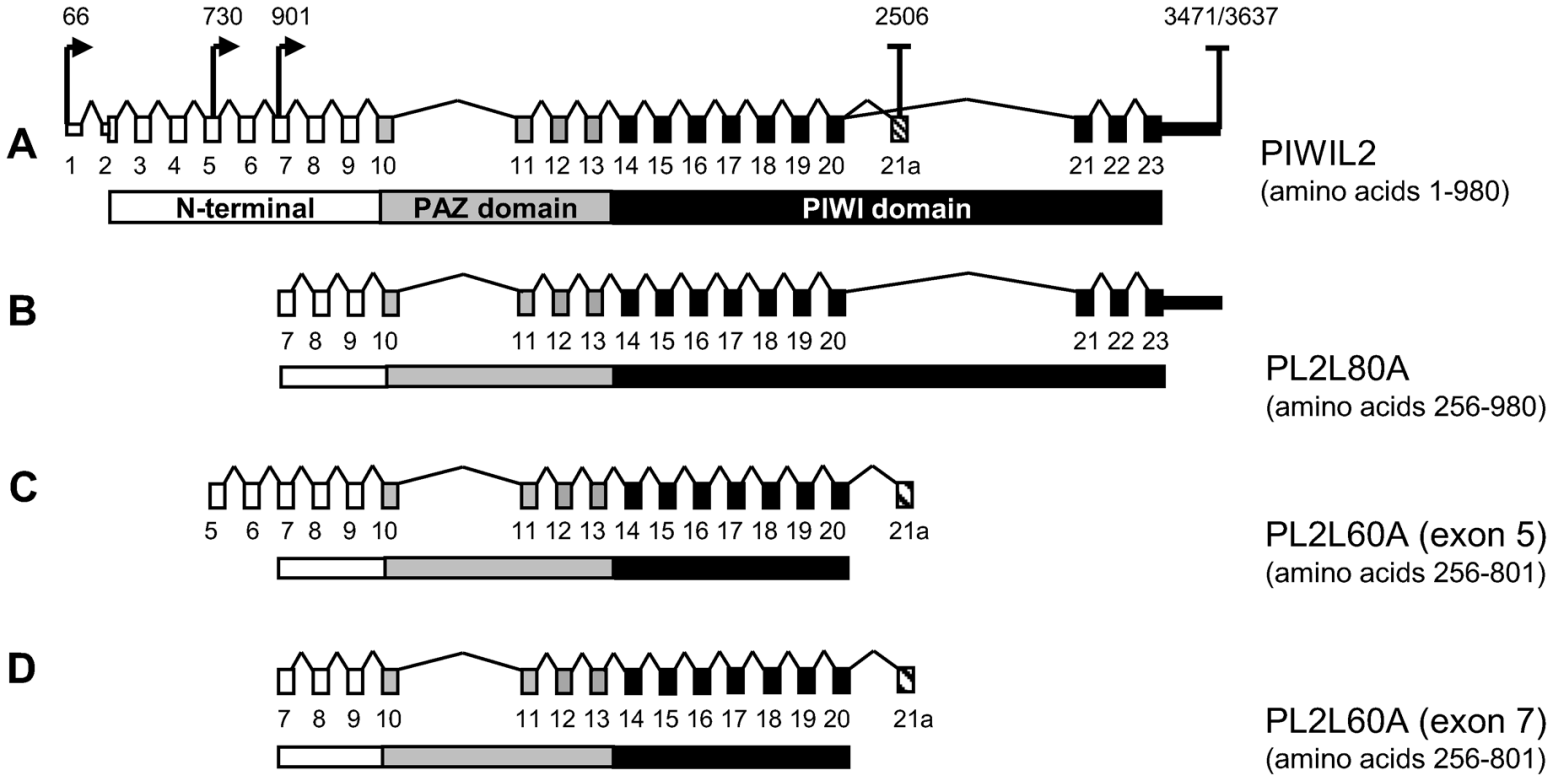
Transcriptional and translational boundaries of PL2L60A and PL2L80A. **A,** Schematic exon/intron structure of the full-length PIWIL2 protein. Small boxes connected by lines represent exons and introns (including the dashed new alternative exon 21a), while long horizontal rectangles correspond to the protein product derived from each mRNA variant. White, grey and black shades refer to N-terminal sequence, PAZ domain and PIWI domain, respectively. Arrows above exons indicate transcription initiation positions, while bars depict polyadenylation sites found in RACE experiments (figures above specify positions in PIWIL2 cDNA NM_018068.3). **B, C, D,** Transcriptional and translational boundaries of PL2L80A in NT2/D1 (panel **B**), PL2L60A in TERA1 cell line (panel **C**) and PL2L60A in testicular tumor samples (panel **D**) with numbers of N-terminal and C-terminal amino acids in each short isoform.

In TERA1, we identified transcription termination sites in exon 15 and in a new alternative 162 bp long exon 21a (in intron 20, Fig. 3A). This new alternative exon was previously described by Ye *et al.* [35] and, since it was only predicted by automated computational analysis (NCBI references XM_942053 and XM_005273551.1), we submitted this transcribed sequence to Genbank EST database (NCBI reference KJ534584). Data from RNA-seq of PolyA + RNA in human embryonic stem cells (H1-hESC) from ENCODE [65], [66] also supports the fact that this genomic sequence is actively transcribed (Fig. S2).

Notably, the new alternative exon 21a is separated from the upstream exon 20 by a 3.5 kbp canonical GU-AG intron. It also contains AAUAAA conservative polyadenylation site and codes for just 3 amino acids followed by an ochre stop codon and a 150 bp 3′-UTR. Additionally, a CTCF binding site just upstream of this alternative exon 21a (ENCODE ChIP-seq data, Fig. S2) is in agreement with the finding that CTCF promotes inclusion of weak exons in the processed mRNA [71].

By consolidating results of Western blot assays along with 5′ and 3′-RACE experiments, promoter activity and *in silico* data we could make conclusions on transcriptional and translational boundaries of PL2L60A in TERA1 and PL2L80A in NT2/D1. Since the transcription start site for PL2L80A in exon 7 (NT2/D1) appears to be validated in 5′-RACE experiments, promoter activity assays and is in accord with the data on chromatin modifications, DNaseI hypersensitivity clusters and transcription factor binding sites from ENCODE, we assume the PL2L80A mRNA to span exons 7 to 23 (Fig. 3B and Table 2, submitted to Genbank as KM434336). Therefore, it lacks the first 255 amino acids spanning the N-terminal domain, though it still retains complete PAZ and PIWI domains.

**Table 2:** Suggested transcriptional and translational boundaries of PL2L60A in TERA1 and PL2L80A in NT2/D1 cell lines.

Cell line	PIWIL2 isoform	Positions of 5′ and 3′ ends of the transcript in PIWIL2 cDNA NM_018068.3 (genomic coordinates in GRCh37/hg19)	Positions of N and C-terminal amino acids (full-length:1–980)	Length and molecular weight
TERA1	PL2L60A (exons 5–21a)	730–2506 (chr8:22140703–22179520)	256–801	546 amino acids, 63 kDa
	PL2L60A (exons 1–15)	66–1856 (chr8:22132875–22167494)	1–568	568 amino acids, 64 kDa
NT2/D1	PL2L80A (exons 7–23)	901-3471/3637 (chr8:22145061-22213423/22213601)	256–980	725 amino acids, 80 kDa

With regard to PL2L60A in TERA1, cumulative results allow us to put forward two hypotheses. Firstly, mRNA for this particular isoform could encompass exons 5 to 21a (Fig. 3C and Table 2) and the resulting protein harbors a complete PAZ domain and truncated N-terminal and PIWI domains. Alternatively, PL2L60A could originate from the region spanning exons 1–15 (Table 2). In this case, this isoform consists of complete N-terminal and PAZ domains and a C-truncated PIWI domain. However, the latter variant might not be detected by the 14–16ex PIWIL2 antibody.

**Figure 4:**
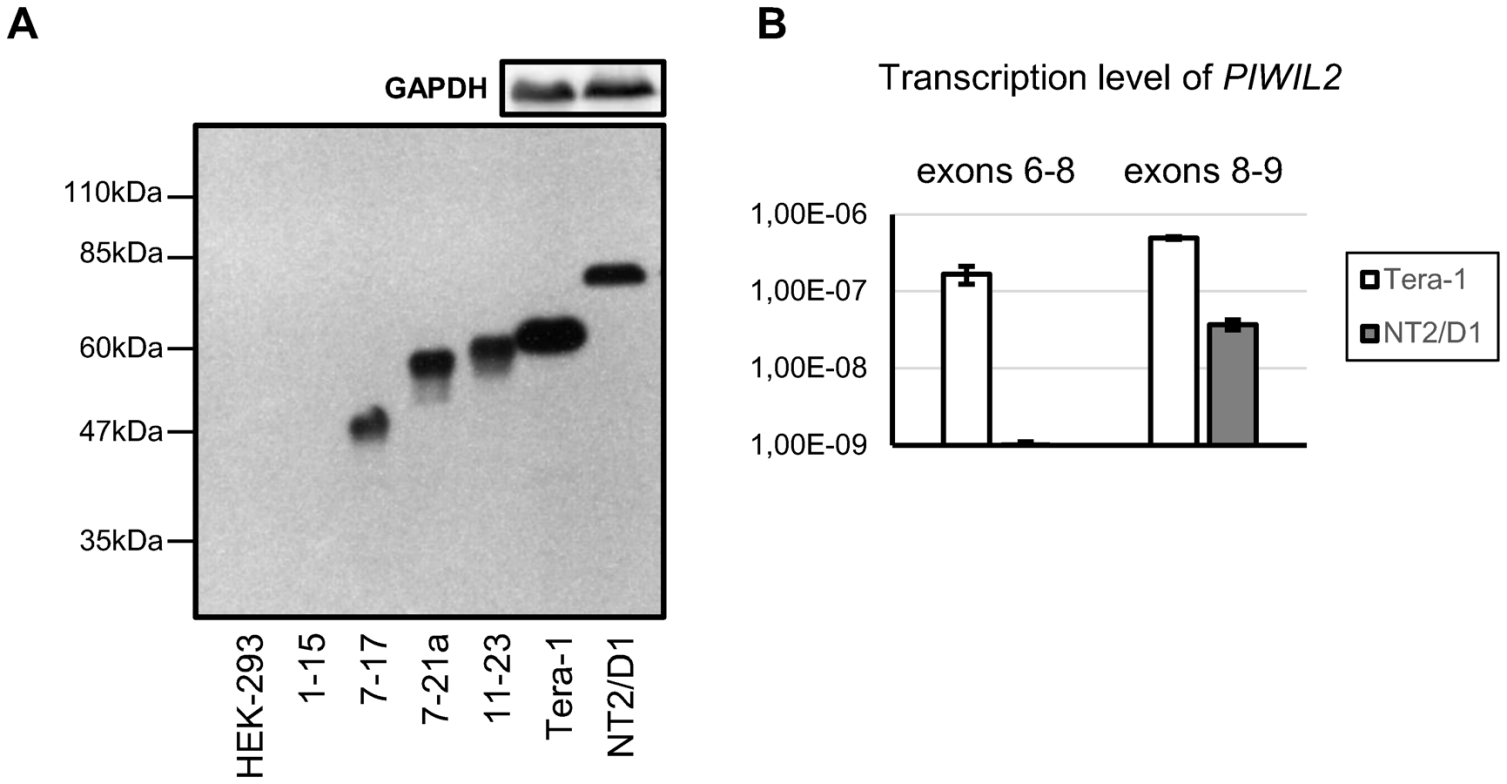
Western blotting with specially constructed PIWIL2 short isoforms and transcription levels of exons downstream of putative promoter regions. **A**, Western blot analysis of TERA1 and NT2/D1 cell lines with the 14–16ex PIWIL2 antibody. Untreated HEK-293 cells and specially constructed PIWIL2 short isoforms transiently expressed in HEK-293 cells were used as controls (table S6): exons 1 to 15 (1–15), exons 7 to 17 (7–17), exons 7 to 21a (7–21a) and exons 11 to 23 (11–23). **B**, Transcription level relative to 18S rRNA of exons downstream of the putative transcription initiation sites.

In order to resolve this ambiguity, we probed specially constructed PIWIL2 short isoforms with the 14–16ex PIWIL2 and full-length PIWIL2 antibodies (Fig. S3A & B and Fig. 4A). In this experiment, short PIWIL2 control isoforms were cloned into pCI vector and transiently expressed in HEK-293 cell line lacking endogenous PIWIL2 expression (Fig. S3A & B). Importantly, these N and C-truncated control variants only interacted with the 14–16ex PIWIL2 antibody if they possessed its full epitope (Fig. S3B and Fig. 4A). Specifically, PIWIL2 control isoform covering exons 1–15 is not detected by the 14–16ex (Fig. S3B and Fig. 4A). Therefore, we could claim that PL2L60A in TERA1 derives from mRNA covering exons 5–21a and is translated from AUG codon in exon 7 (Fig. 3C).

Remarkably, both PL2L60A in TERA1 and PL2L80A in NT2/D1 share the same AUG start codon in exon 7, though their mRNAs are transcribed from different start sites in exons 5 and 7, respectively (Fig. 3B & C). To confirm the transcription start sites of these two isoforms, we performed RT-qPCR on mRNA from the two cell lines with primers for exons immediately downstream of the both transcription start sites: exons 6–8 for PL2L60A in TERA1 and exons 8–9 for PL2L80A in NT2/D1. In order to be able to compare two different primer pairs, we normalized for their PCR efficiency using cloned PIWIL2 cDNA sequence. In line with the previous findings, transcription in TERA1 does start before exon 6, while in NT2/D1 it is only initiated after exon 7 (Fig. 4B).

### Both mRNA and protein levels of PL2L60A expression in TGCTs of various subtypes could serve as a marker of their differentiation stage

**Figure 5:**
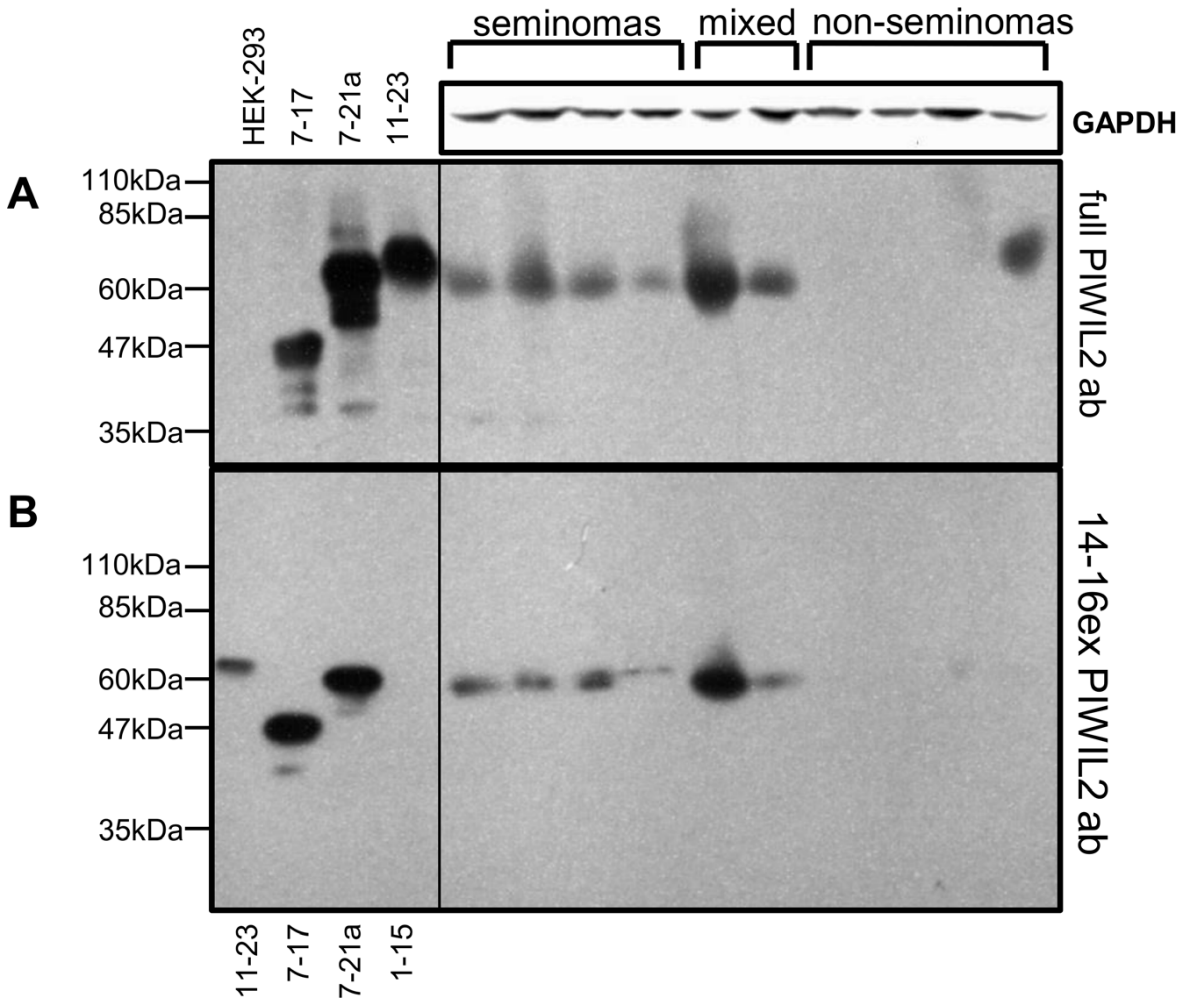
Western blot analysis of testicular tumors. Western blot analysis of 4 seminomas, 2 mixed and 4 nonseminomatous tumors with antibodies specific to the full-length PIWIL2 (panel **A**), exons 14–16 (panel **B**). Untreated HEK-293 cells and specially constructed PIWIL2 isoforms transiently expressed in HEK-293 cells were used as controls (table S6): exons 1 to 15 (1–15), exons 7 to 17 (7–17), exons 7 to 21a (7–21a) and exons 11 to 23 (11–23).

In recent years, many research groups have managed to demonstrate overexpression of PIWIL2 in various human cancers. Therefore, we extended our study to several TGCTs samples (4 undifferentiated seminomas, 4 differentiated nonseminomatous tumors and 2 mixed tumors) and examined them for presence of different PIWIL2 isoforms using two antibodies: specific to exons 14–16 and the full-length protein. Interestingly, almost all samples expressed predominantly PL2L60A, though at different levels (Fig. 5A & B). In particular, PL2L60A protein expression was higher in undifferentiated seminomas and mixed tumors and lower in differentiated nonseminomatous tumors (Fig. 5A & B).

In order to confirm specificity of PL2L60A expression to undifferentiated seminomas, we expanded the number of samples to 42 (12 seminomas, 12 nonseminomatous tumors and 18 mixed tumors) and performed RT-qPCR with primers for exons 6–8 (in case transcription starts from exon 5) and 8–9 (in case transcription starts from exon 7). At the same time, to provide more detailed profiling information on the level of differentiation of the samples under investigation, we analyzed mRNA level of the transcription factor OCT4 (*POU5F1*), which is proved to be an embryonic stem cell marker actively transcribed in undifferentiated seminomas and embryonic carcinomas [72], [73].

**Figure 6:**
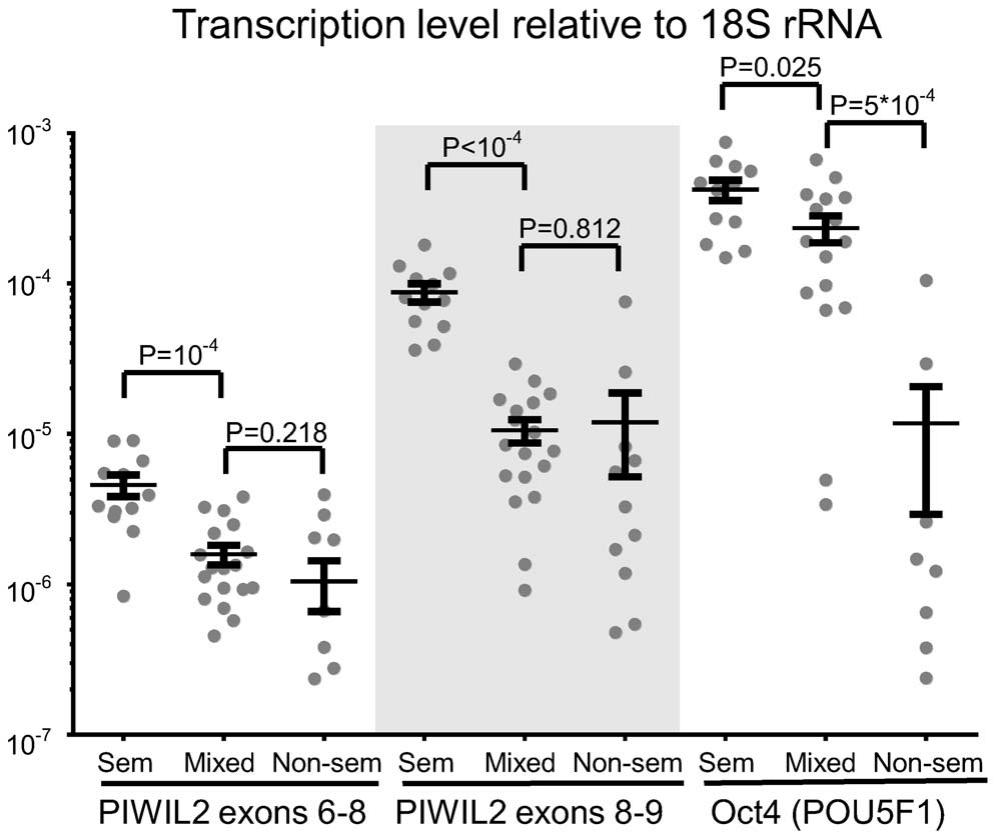
Transcription level of PL2L60A and OCT4 (*POU5F1*) in testicular tumors of various differentiation subtypes. Transcription levels relative to 18S rRNA of PIWIL2 exons 6–8 and exons 8–9, as well as OCT4 (*POU5F1*) in seminomas (SEM), mixed (MIXED) and nonseminomatous (NON-SEM) tumors. Mean value, standard error of mean interval for each group and P-values in two-sample *t*-test are calculated and presented. 4 specimens of nonseminomatous tumors with undetectable level of OCT4 mRNA and 4 specimens of nonseminomatous tumors with transcription level of PIWIL2 exons 6–8 below 10^−7^ are not shown.

OCT4 mRNA expression level was shown to be significantly higher in seminomas, slightly less in mixed tumors and almost undetectable in nonseminomatous tumors (Fig. 6). Almost the same pattern was followed by PL2L60A, where seminomas displayed the highest level of its mRNA, whereas mixed samples and nonseminomatous cancers exhibited a lower degree of its expression (Fig. 6).

Importantly, mRNA level difference for exons 6–8 between seminomas, mixed samples and nonseminomatous tumors was about 4-fold, unlike exons 8–9 which exhibited around 10-fold shift (Fig. 6). Additionally, average values of mRNA abundance for exons 6–8 were about 10 times lower than for exons 8–9 in seminomas, which expressed PL2L60A protein variant. Therefore, we might suggest that transcription of PL2L60A mRNA in seminomas starts from exon 7, unlike TERA1 where it is initiated in exon 5. However, in both cases, the same start AUG codon in exon 7 is utilized. The relatively low level of mRNA spanning exons 6–8 could represent cryptic transcription [74]–[76] or, alternatively, transcription from intragenic regulatory elements [77]–[80].

### Retinoic acid induced differentiation confirms specificity of PIWIL2 short isoforms expression for undifferentiated tumor subtypes

In order to validate previous findings of the correlation between tumor differentiation subtype and PIWIL2 short isoforms expression, we used an *in vitro* cell line model experiment. Here, we tracked the changes of expression of PL2L60A (from exon 5 in TERA1, malignant embryonal carcinoma) and PL2L80A (from exon 7 in NT2/D1, malignant *pluripotent* embryonal carcinoma) during retinoic acid induced differentiation [62], [63], [81], [82]. Transcription level of *NANOG* mRNA was used as a control marker, since it is more indicative of early stages of differentiation in NT2/D1 [73], [83], [84].

**Figure 7:**
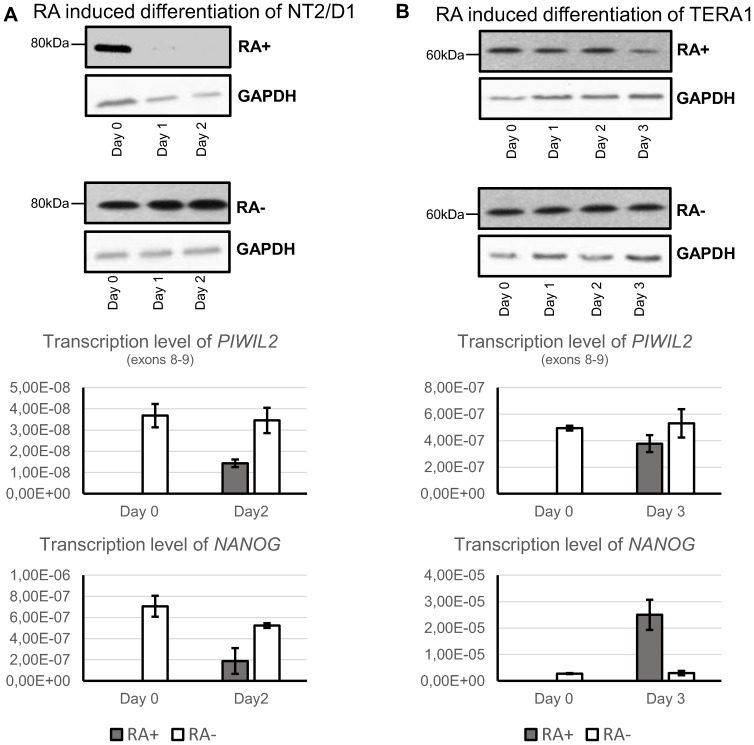
Retinoic acid induced differentiation of NT2/D1 and TERA1 cell lines. Western blotting with 14–16ex PIWIL2 antibody and transcription levels of *PIWIL2* (exons 8–9), *NANOG* and OCT4 (*POU5F1*) relative to 18S rRNA in NT2/D1 (**A**) and TERA1 (**B**) cell lines. (RA+) - experiment with retinoic acid treatment, (RA-) - controls with no treatment.

In NT2/D1, PL2L80A mRNA level significantly decreases and protein isoform expression is undetectable on Day 2 after retinoic acid induction (Fig. 7A). Therefore, these data confirm the assumption that promoter region around exon 7 for PL2L80A (in NT2/D1) and for PL2L60A (in seminomas) is more active in undifferentiated testicular tumors and pluripotent carcinoma cell line and its activity goes down in the course of differentiation.

Unlike NT2/D1, TERA1 cell line (Fig. 7B) demonstrated its negligible capacity for cell differentiation, which was previously shown by other groups [85], [86].

## Discussion

The volume of data on expression profiles of PIWIL2 in different cancers suggests its implication in carcinogenesis and has prompted many research groups to propose it as an oncological marker. There has been a significant body of statistics collected on the presence of PIWIL2 protein and its mRNA transcript in various types of cancers including breast tumors [21], [22], acute myeloid leukemia [87], papillary thyroid carcinoma [26], as well as colorectal [31], colon [24], [27], gastric [25], ovarian [32] and cervical cancers [23], [28].

PIWIL2 was demonstrated to impact the development of precancerous stem cells into cancers [30], [88]. Additionally, its ectopic expression in mouse embryonic fibroblasts was also shown to influence their migration and invasion characteristics [89]. Furthermore, the knockdown of PIWIL2 in SW620 and SW480 cell lines derived from colon cancer significantly reduced invasive proliferation [24]. Other groups of researchers have provided a certain amount of evidence on PIWIL2 involvement in tumorigenesis by suppressing p53 through STAT3/c-Src [34], as well as influence of PIWIL2 on upregulation of STAT3, Bcl2 and nuclear expression of NF-kB [35] and activation of STAT3/Bcl-X(L) pathway [36]. Among others, PIWIL2 is also shown to be involved, to some extent, in TGF-beta signaling [33] and DNA damage repair and concomitant chromatin modifications [29], [90], which could also contribute to neoplasia.

Also, the fact that PIWIL2 is expressed as a set of its isoforms increases the number of players to be taken into account [35]. Moreover, Ye *et al.* [35] found the 60 kDa variant (PL2L60) to be predominantly present in various types of human and mouse tumor cells.

In this research, we made an effort to shed more light on the incidence of short isoforms of PIWIL2 in a range of TGCTs and cell lines related to them. For that purpose, we used a commercially available antibody specific to exons 14–16 of PIWIL2 protein (14–16ex PIWIL2 antibody, HPA029345, Sigma, USA). We validated the specificity of this antibody using both siRNA assay and a set of specially constructed short PIWIL2 isoforms as additional controls. Because we and Ye *et al.* [35] used different antibodies, straightforward comparison of our results was inappropriate. Therefore, we termed all short PIWIL2 isoforms discovered in our study differently from Ye *et al.* [35].

Curiously enough, using 14–16ex PIWIL2 antibody we could detect expression of PIWIL2 short isoforms only in a few cell lines examined: 60 kDa isoform (PL2L60A) in TERA1, T47D and Raji, 80 kDa isoform (PL2L80A) in NT2/D1 and the full-length PIWIL2 in Daudi cells (Fig. 1A).

Further, using RACE experiments and promoter activity luciferase assay, we ascertained and validated the promoter regions for PIWIL2 isoforms in testicular cancer cell lines: for PL2L60A upstream of exon 5 in TERA1 and for PL2L80A upstream of exon 7 in NT2/D1. These alternative transcription initiation sites were supported by the data mined *in silico* on chromatin modifications, DNaseI hypersensitivity clusters and transcription factor binding sites. An important consideration here is that both alternative promoters in exons 5 and 7 initiate transcription of mRNAs which share the same AUG start codon in exon 7.

Next, through combining the data from RACE experiments, promoter activity assays, RT-qPCRs and Western blot analyses with specially constructed PIWIL2 short isoforms, we were able to make conclusions on the transcriptional boundaries of PL2L60A in TERA1 and PL2L80A in NT2/D1 cell lines. Specifically, we could suggest that PL2L80A mRNA (NT2/D1) covers exons 7 to 23 (Fig. 3B) and the resulting protein lacks a significant part of N-terminal sequence. On the other hand, PL2L60A mRNA (TERA1) spans exons 5 to 21a (Fig. 3C) and, thus, includes a functional PAZ domain but also harbors incomplete N-terminal and PIWI domains.

Consequently, PL2L60A and PL2L80A are bound to possess properties distinct from each other and the full-length protein. In particular, the absence of the evolutionarily conserved symmetrical dimethylarginines in N-terminal sequence [43], [44] could deprive both PL2L80A and PL2L60A of ability to bind TUDOR domain containing proteins which, in turn, may prevent this isoform from participating in piRNA/PIWI machinery [41], [42], [91]. Similarly, the lack of a functional RNase H PIWI domain in PL2L60A should invariably disengage it from the conventional functions of PIWIL2. At the same time, there must be some biologically significant properties inherent to PL2L60A, since it is expressed in many TGCTs (see below). A similar context is described in Drosophila, where *piwi^Nt^* mutation removing nuclear localization signal from PIWI protein leads to the impaired transposon silencing function but does not affect the maintenance of germ stem cells [13].

Furthermore, using a collection of 42 TGCT samples comprising both undifferentiated seminomas and differentiated subtypes of TGCTs, we managed to demonstrate that both mRNA and protein levels of PL2L60A expression are significantly higher in undifferentiated seminomas. Conversely, in mixed and differentiated samples PL2L60A mRNA and protein levels are substantially decreased. Therefore, PL2L60A expression pattern might be proposed as a marker to distinguish between variously differentiated TGCT subtypes, since different prognosis and treatment are characteristic to these tumors in clinical practice [92]–[94]. Moreover, PL2L60A expression profile could complement the data on expression of other proteins exhibiting correlation with TGCT differentiation stage, such as HMGA1/2 and estrogen receptor β [95], [96].

To confirm changes of PL2L60A expression in an *in vitro* differentiation model, we used retinoic acid induced differentiation of NT2/D1. Consistent with the results in TGCTs, PL2L80A expression of both mRNA and protein decreased in NT2/D1 after the induction with RA. The outcome clearly reveals a certain degree of interrelation between differentiation stage and short isoforms expression pattern, which supports our findings in TGCTs profiling and is consistent with the results of other research groups [97], [98].

In conclusion, the involvement of PIWIL2 in carcinogenesis should not be considered as straightforward as it may seem. The discovery of multiple PIWIL2 isoforms and their non-uniform incidence among tumors and normal tissues makes the case for more careful attention to the PIWIL2 variants profile rather than just the total set of translation products of the gene, let alone the full-length protein. One might assert that PIWIL2 isoforms bearing various combinations of domains are bound to be of biological significance for such malconditions as infertility [99] and tumors [19].

## Supporting Information

Figure S1 **UCSC Genome Browser view of PIWIL2 genomic sequence for exons 1–13 in GRCh37/hg19 human genome assembly.** The browser view demonstrates association of alternative transcription start sites in exons 5 and 7 with genomic features characteristic for active promoter and regulatory regions: chromatin modifications (ChIP-seq for H3K4Me1, H3K4Me3, H3K27Ac in 7 cell lines from ENCODE), DNaseI hypersensitivity clusters (in 125 cell lines from ENCODE) and Transcription Factor Binding Sites (ChIP-seq for 161 factors in 91 cell lines from ENCODE).
(TIFF)

Figure S2 **UCSC Genome Browser view of PIWIL2 genomic sequence for exons 17–20 in GRCh37/hg19 human genome assembly.** The position of the alternative exon 21a is shown to coincide with RNA-seq data from PolyA + RNA in human embryonic stem cells (H1-hESC) from ENCODE. ChIP-seq data from ENCODE demonstrate presence of a CTCF binding just upstream this alternative exon, which could promote inclusion of exons while splicing pre-mRNA [71].
(TIFF)

Figure S3 **Western blot analysis of specially constructed PIWIL2 control isoforms.** Western blot analysis of untreated HEK-293 cells and HEK-293 cells transiently transfected with specially constructed PIWIL2 control isoforms (table S6) with antibodies specific to the full-length PIWIL2 (panel **A**) and exons 14-16 (panel **B**): exons 1 to 15 (1–15), exons 1 to 21a (1–21a), exons 7 to 14 (7–14), exons 7 to 17 (7–17), exons 7 to 21a (7–21a), exons 11 to 23 (11–23) and the full-length PIWIL2 (1–23).
(TIFF)

Table S1 **Antibodies used in Western blot analyses.**
(DOCX)

Table S2 **Primers used in 5′-RACE experiments with TERA1 and NT2/D1 cell lines.**
(DOCX)

Table S3 **Primers used in 3′-RACE experiments with TERA1 and NT2/D1 cell lines.**
(DOCX)

Table S4 **Primer sequences for mRNA profiling assays.**
(DOCX)

Table S5 **Cloning genomic regions upstream of putative transcription initiation sites for promoter activity assays.**
(DOCX)

Table S6 **Primers for cloning control PIWIL2 shorter isoforms.**
(DOCX)
