# Supplementary figures and images for: Expression Profiles of PIWIL2 Short Isoforms Differ in Testicular Germ Cell Tumors of Various Differentiation Subtypes
Source: PLoS One. 2014 Nov 10;9(11):e112528. doi: 10.1371/journal.pone.0112528 (PMC4226551; doi:10.1371/journal.pone.0112528)

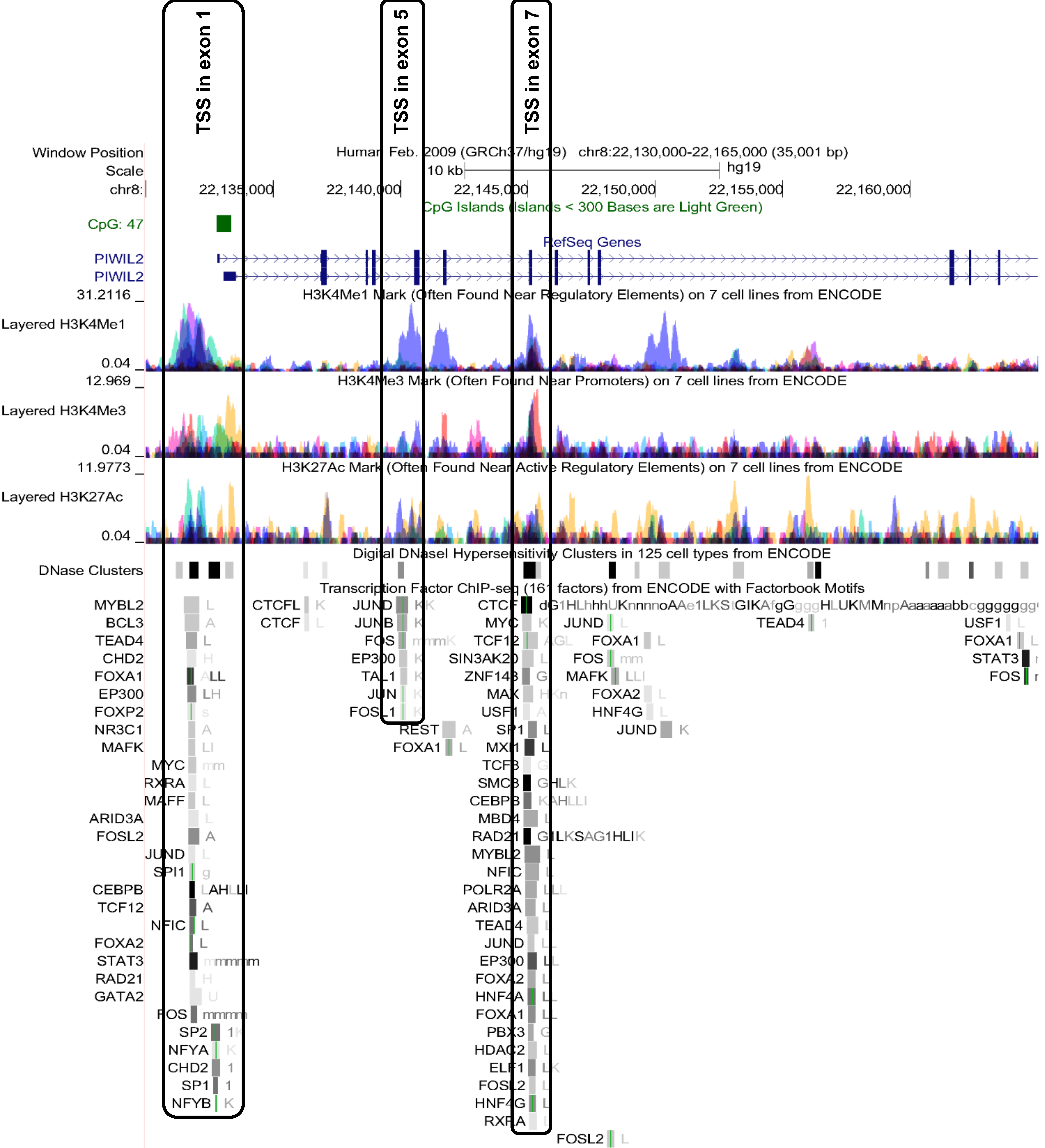

Supplement: Figure S1 — UCSC Genome Browser view of PIWIL2 genomic sequence for exons 1–13 in GRCh37/hg19 human genome assembly. The browser view demonstrates association of alternative transcription start sites in exons 5 and 7 with genomic features characteristic for active promoter and regulatory regions: chromatin modifications (ChIP-seq for H3K4Me1, H3K4Me3, H3K27Ac in 7 cell lines from ENCODE), DNaseI hypersensitivity clusters (in 125 cell lines from ENCODE) and Transcription Factor Binding Sites (ChIP-seq for 161 factors in 91 cell lines from ENCODE). (TIFF) [file pone.0112528.s001.tiff]

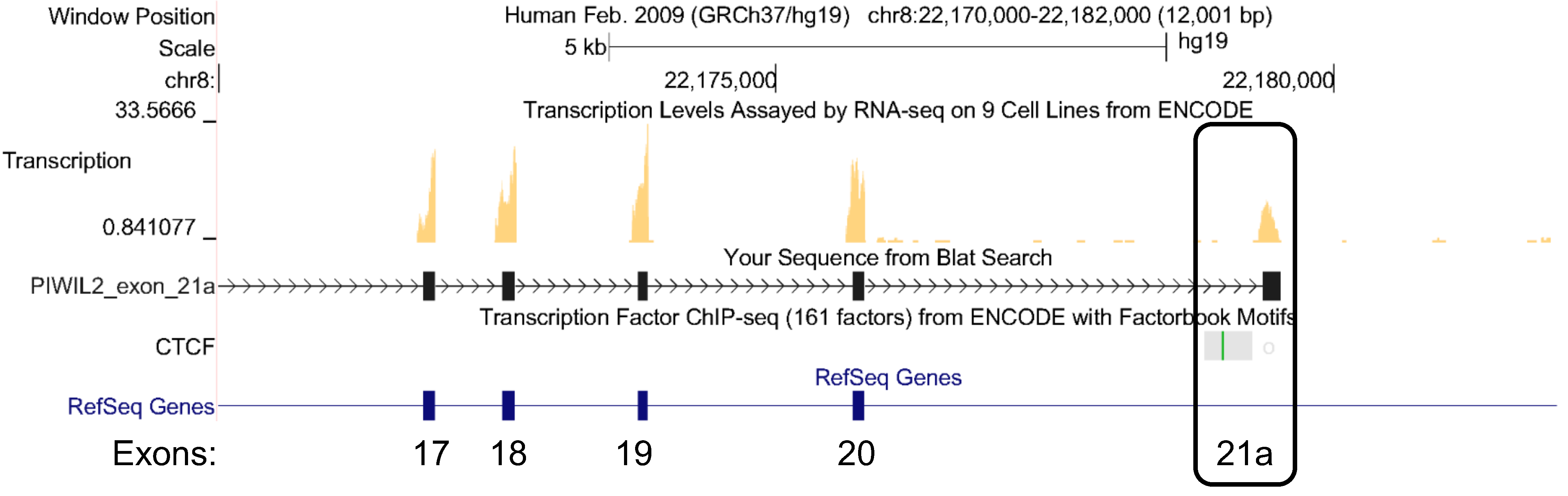

Supplement: Figure S2 — UCSC Genome Browser view of PIWIL2 genomic sequence for exons 17–20 in GRCh37/hg19 human genome assembly. The position of the alternative exon 21a is shown to coincide with RNA-seq data from PolyA + RNA in human embryonic stem cells (H1-hESC) from ENCODE. ChIP-seq data from ENCODE demonstrate presence of a CTCF binding just upstream this alternative exon, which could promote inclusion of exons while splicing pre-mRNA [71]. (TIFF) [file pone.0112528.s002.tiff]
