# Supplementary material for: Expression Profiles of PIWIL2 Short Isoforms Differ in Testicular Germ Cell Tumors of Various Differentiation Subtypes
Source: PLoS One. 2014 Nov 10;9(11):e112528. doi: 10.1371/journal.pone.0112528 (PMC4226551; doi:10.1371/journal.pone.0112528)
Supplement: Table S1 — Antibodies used in Western blot analyses. (DOCX) [file pone.0112528.s004.docx]

**Table S1.** Antibodies used in Western blot analyses.

| Antibody (antigen) | Producer, catalogue number | Host species, poly/monoclonal | Dilution, final concentration | Antibody Registry ID |
| --- | --- | --- | --- | --- |
| Human PIWIL2 (exons 14-16, amino acids 522-664) | Sigma-Aldrich (USA), HPA029345 | Rabbit polyclonal | 1:100, 1 mkg/ml | AB_10602117, AB_10549059 |
| Human PIWIL2 (full length, amino acids 1-973) | Abcam (UK), ab169698 | Mouse polyclonal | 1:1000, 1 mkg/ml | n/a |
| Human GAPDH | Santa Cruz Biotechnology (USA), sc-47724 | Mouse monoclonal | 1:1000, 0.2 mkg/ml | AB_627678 |
