# Supplementary material for: Expression Profiles of PIWIL2 Short Isoforms Differ in Testicular Germ Cell Tumors of Various Differentiation Subtypes
Source: PLoS One. 2014 Nov 10;9(11):e112528. doi: 10.1371/journal.pone.0112528 (PMC4226551; doi:10.1371/journal.pone.0112528)
Supplement: Table S2 — Primers used in 5′-RACE experiments with TERA1 and NT2/D1 cell lines. (DOCX) [file pone.0112528.s005.docx]

**Table S2.** Primers used in 5’-RACE experiments with TERA1 and NT2/D1 cell lines.

| Primer set | cDNA synthesis primer | 1^st^ round PCR primer | 2^nd^ round PCR primer |
| --- | --- | --- | --- |
| Exons 5,3 | ACGCATCTGAACTCCCTCTTC | CTCGGAACATGGAGACCAAA | CTCGGAACATGGAGACCAAA |
| Exons 9,8,5 | GTTTCTCCCCACAAGCTTCA | CCAGGATCTTTGTCATCTGAATC | ACGCATCTGAACTCCCTCTTC |
| Exons 12,11,9 | GGTCCTCTTCCTTAACTGTGATCC | ACACTCATCCTGGAAGTGTTCTTT | GTTTCTCCCCACAAGCTTCA |
| Exons 16,15,14 | GGATTGAATGGTTCTGACATAAGTC | TTCCATTGGCAGAACACG | AGCAAGCATTCCAAAGCA |
| Exons 18,17,16 | CCACCCAATTTACAGTTAATCTG | CACAGCACAGCTTCTTGATG | GGATTGAATGGTTCTGACATAAGTC |
| Exons 22,21,20 | GAACACAGACATAATGCGTAG | CAAAGTTCTGAGGAGCAGCC | AGGCTGTCCACAATCTCCTG |
