# Supplementary material for: Expression Profiles of PIWIL2 Short Isoforms Differ in Testicular Germ Cell Tumors of Various Differentiation Subtypes
Source: PLoS One. 2014 Nov 10;9(11):e112528. doi: 10.1371/journal.pone.0112528 (PMC4226551; doi:10.1371/journal.pone.0112528)
Supplement: Table S3 — Primers used in 3′-RACE experiments with TERA1 and NT2/D1 cell lines. (DOCX) [file pone.0112528.s006.docx]

**Table S3.** Primers used in 3’-RACE experiments with TERA1 and NT2/D1 cell lines.

| Primer set | 1^st^ round PCR primer | 2^nd^ round PCR primer |
| --- | --- | --- |
| Exons 2,4 | CAGGCAGAGGCCATGTATTT | CAAGGACAGGGAGGAACTCTC |
| Exons 6,8 | CAGTGTCATAATGAAGCAGTTTATC | TGACCTGTGCATTCCCTTCT |
| Exons 10,11 | TTCGGAATGACTGTGTGCTG | GAGATCACATTCTTGGAATACTACAG |
| Exon 12 | TGGGATCACAGTTAAGGAAGAG | GTGAGAGACAGGATAATCATGGG |
| Exons 14,15 | GCAAATCAATCTGAGCCCC | TCGTTTATCACATCTCAGGAAC |
| Exons 16,17 | CCTGGGTTGAACTAAAGGATG | CATCAAGAAGCTGTGCTGTG |
| Exons 18,20 | TCGAACCATTGGTCAGC | GATTGTGGACAGCCTGAAGC |
| Exons 21,22 | GAACCACTGTCTACCAGAGAAG | GGATTTCTATCTTCTTGCCCATC |
| Exons 22,23 | GGATTTCTATCTTCTTGCCCATC | ACTTTCAAACTGTGCCACATGTAC |
