# Supplementary material for: Expression Profiles of PIWIL2 Short Isoforms Differ in Testicular Germ Cell Tumors of Various Differentiation Subtypes
Source: PLoS One. 2014 Nov 10;9(11):e112528. doi: 10.1371/journal.pone.0112528 (PMC4226551; doi:10.1371/journal.pone.0112528)
Supplement: Table S4 — Primer sequences for mRNA profiling assays. (DOCX) [file pone.0112528.s007.docx]

**Table S4.** Primer sequences for mRNA profiling assays.

| Primer pair | Sequences | Product size, bp |
| --- | --- | --- |
| *PIWIL2* exons 2-23 | CAGGCAGAGGCCATGTATTT and GCTGTTACTCAGAAACTTG | 2980 |
| *PIWIL2* exons 6-8 | CAGTGTCATAATGAAGCAGTTTATC and CCAGGATCTTTGTCATCTGAATC | 238 |
| *PIWIL2* exons 8-9 | TGACCTGTGCATTCCCTTCT and GTTTCTCCCCACAAGCTTCA | 79 |
| *NANOG* | GCTGAAGAATAGCAATGGTG and AGTCGGGTTCACCAGGCA | 97 |
| OCT4 (*POU5F1)* | TGCAGCAGATCAGCCACAT and TAGTCGCTGCTTGATCGCTT | 106 |
| 18S rRNA | CGCGGTTCTATTTTGTTGGT and ATGCCAGAGTCTCGTTCGTT | 521 |
