# Supplementary material for: Expression Profiles of PIWIL2 Short Isoforms Differ in Testicular Germ Cell Tumors of Various Differentiation Subtypes
Source: PLoS One. 2014 Nov 10;9(11):e112528. doi: 10.1371/journal.pone.0112528 (PMC4226551; doi:10.1371/journal.pone.0112528)
Supplement: Table S5 — Cloning genomic regions upstream of putative transcription initiation sites for promoter activity assays. (DOCX) [file pone.0112528.s008.docx]

**Table S5.** Cloning genomic regions upstream of putative transcription initiation sites for promoter activity assays.

| Primer pair | Primer sequences | Product Size, bp | Genomic coordinates in GRCh37/hg19 |
| --- | --- | --- | --- |
| exon 1 | CTAGCTAGCGGGCAAGTCAATTTCAACACGC and CTAAAGCTTCACAGCCCTGCCAGGGGT | 665 | chr8:22132542-22133206 |
| exon 4 | CTAGCTAGCGTTTATAAAATGTTTCCCTTTATTCTTAT and CTAGCTAGCTTAACAGCTGCTGACTACTCTTGA | 691 | chr8:22138495-22139185 |
| exon 5 | CTAGCTAGCGGTGATTCTTTAATAACGTTGAGTC and CTAGCTAGCTCCTTGTGTTCCACAGTCAGG | 748 | chr8:22140003-22140750 |
| exon 7 | CTAGCTAGCGGAGCTGAGCACATACAACAGG and CTAGCTAGCCACAGCATGACAACTACGATTCAG | 2001 | chr8:22143900-22145900 |
| exon 11 | CTAGCTAGCGAGTATTTCAAAGTACGAAATTT and CTAGCTAGCTAGTATTCCAAGAATGTGATCTCTTT | 845 | chr8:22160875-22161719 |
