# Supplementary material for: Expression Profiles of PIWIL2 Short Isoforms Differ in Testicular Germ Cell Tumors of Various Differentiation Subtypes
Source: PLoS One. 2014 Nov 10;9(11):e112528. doi: 10.1371/journal.pone.0112528 (PMC4226551; doi:10.1371/journal.pone.0112528)
Supplement: Table S6 — Primers for cloning control PIWIL2 shorter isoforms. (DOCX) [file pone.0112528.s009.docx]

**Table S6.** Primers for cloning control PIWIL2 shorter isoforms.

| PIWIL2 variant | Primer pair sequence (restriction enzyme recognition site) | PIWIL2 isoform cDNA boundaries (NM_018068.3), genomic coordinates in GRCh37/hg19 | PCR product size/Protein molecular weight |
| --- | --- | --- | --- |
| Exons 1-15 | TATAGAATTCACCATGGATCCTTTCCGACCATCGTT (EcoRI) and TATAGCGGCCGCTTACATTGGCAGAACACGTCC (NotI) | 150-1862, chr8:22136900-22167500 | 1713bp/64kDa |
| Exons 1-21a | TATAGAATTCACCATGGATCCTTTCCGACCATCGTT (EcoRI) and TATAGCGGCCGCTTACTGCCATTCCTCATAAAACTTTTTTAAGG (NotI) | 150-2564, chr8:22136900-22179374 | 2415bp/90kDa |
| Exons 7-14 | TATAGAATTCACCATGAGGTTCGGCATGTTGAA (EcoRI) and TATAGCGGCCGCTTACTTTTGCAGACGGAGCC (NotI) | 915-1826, chr8:22145064-22165578 | 912bp/35kDa |
| Exons 7-17 | TATAGAATTCACCATGAGGTTCGGCATGTTGAA (EcoRI) and TATAGCGGCCGCTTACTGGGAGGGCACTGGG (NotI) | 915-2243, chr8:22145064-22172545 | 1329bp/51kDa |
| Exons 7-21a | TATAGAATTCACCATGAGGTTCGGCATGTTGAA (EcoRI) and TATAGCGGCCGCTTACTGCCATTCCTCATAAAACTTTTTTAAGG (NotI) | 915-2564, chr8:22145064-22179374 | 1650bp/63kDa |
| Exons 11-23 | TATAGAATTCACCATGCATGCCATTTATCAGC (EcoRI) and TATAGCGGCCGCTCAGCTGTTACTCAGAAACTTG (NotI) | 1329-3157, chr8:22147859-22213213 | 1829bp/67kDa |
| Exons 1-23 (full-length) | TATAGAATTCACCATGGATCCTTTCCGACCATCGTT (EcoRI) and TATAGCGGCCGCTCAGCTGTTACTCAGAAACTTG (NotI) | 150-3157, chr8: 22136900-22213213 | 3008bp/110kDa |
